# Behavioral Animal Models and Neural-Circuit Framework of Depressive Disorder

**DOI:** 10.1007/s12264-024-01270-7

**Published:** 2024-08-09

**Authors:** Xiangyun Tian, Scott J. Russo, Long Li

**Affiliations:** 1 State Key Laboratory of Brain and Cognitive Science, Institute of Biophysics, Chinese Academy of Sciences, Beijing, 100101 China; 2 University of the Chinese Academy of Sciences, Beijing, 100049 China; 3 Nash Family Department of Neuroscience, Icahn School of Medicine at Mount Sinai, New York, NY 10029 USA; 4 Friedman Brain Institute, Icahn School of Medicine at Mount Sinai, New York, NY 10029 USA

**Keywords:** Depression, Animal models, Stress, Neural circuits

## Abstract

Depressive disorder is a chronic, recurring, and potentially life-endangering neuropsychiatric disease. According to a report by the World Health Organization, the global population suffering from depression is experiencing a significant annual increase. Despite its prevalence and considerable impact on people, little is known about its pathogenesis. One major reason is the scarcity of reliable animal models due to the absence of consensus on the pathology and etiology of depression. Furthermore, the neural circuit mechanism of depression induced by various factors is particularly complex. Considering the variability in depressive behavior patterns and neurobiological mechanisms among different animal models of depression, a comparison between the neural circuits of depression induced by various factors is essential for its treatment. In this review, we mainly summarize the most widely used behavioral animal models and neural circuits under different triggers of depression, aiming to provide a theoretical basis for depression prevention.

## Overview of Depressive Disorder

Depressive disorder is one of the most common neuropsychiatric disorders across the world. It affects interpersonal relationships, social life, and one’s sense of self-worth, leading to severe dysfunction [1]. The American Psychiatric Association’s *Diagnostic Statistical Manual of Mental Disorders, Fifth Edition* classifies depressive disorders as the following: major depressive disorder (MDD), persistent depressive disorder, disruptive mood dysregulation disorder, premenstrual dysphoric disorder, and depressive disorder due to another medical condition. MDD is also referred to as depression [2]; its development may be influenced by genetic factors, situational stress, medical conditions, adverse emotional experiences, particularly those in childhood, and individual resilience. It is characterized by persistent sadness, loss of interest or pleasure, low energy, changes in food consumption, worse appetite and sleep, and even suicidal thoughts, disrupting daily activities and psychosocial functions. In 2010, MDD was the second most significant contributor to global disability, accounting for 8.2% of the global years lived with disability (YLD) [3]. In 2016, the Global Burden of Diseases, Injuries, and Risk Factors Study revealed that depression was responsible for 34.1 million YLDs, positioning it as the fifth leading cause of YLD [4]. Recently, WHO has predicted that depression will be the leading cause of disease burden worldwide by 2030 [3].

**Fig. 1:**
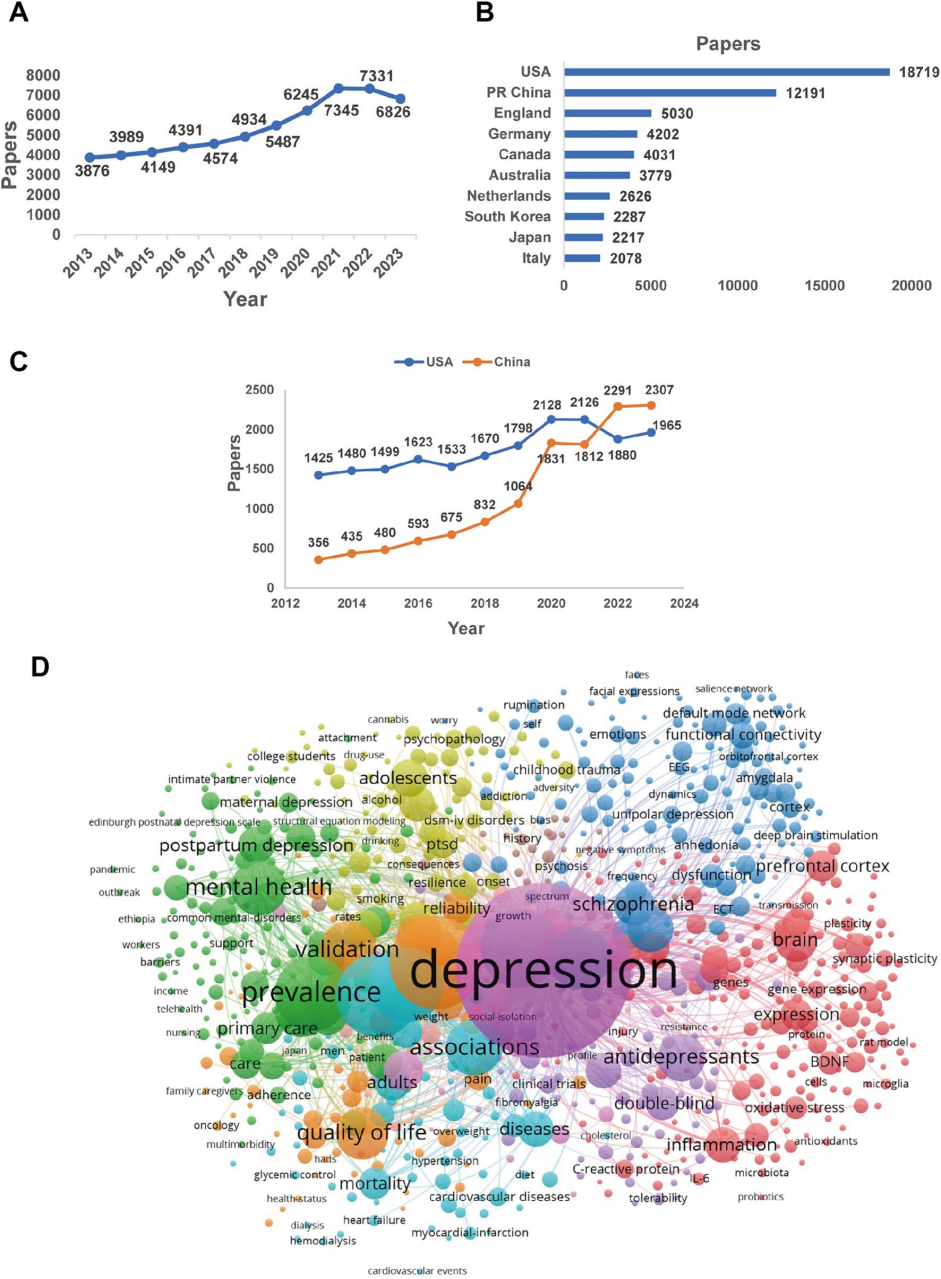
Analysis of published papers around the world from 2013 to 2023 on depressive disorder. **A** The total number of papers from a search of the SCI-Expanded Web of Science database (search strategy: (TI = (depression$) or ts = (“major depressive disorder$”)) and py = (2013–2023)]. **B** The top 10 countries publishing on the topic of depressive disorder. **C** Comparison of papers in China and the USA. **D** Hot topics on depressive disorder research and keyword co-occurrence analysis were conducted using VOSviewer software.

MDD exerts a severe social and economic burden globally. The annual economic cost of MDD in the United States alone is an astonishing $70 billion in medical expenses, lost productivity, and other costs [5]. Many researchers have conducted a series of animal experiments and clinical studies on the pathogenesis and effective therapies for depression. In the past decade, there has been a rise in the total count of research papers published globally on depression almost every year, as shown in Fig. 1A. Searching the SCI-Expanded Web of Science database [2], we found a total of 59,156 articles published in the depression field from 2013 to 2023 (search strategy: (TI = (depression$) or ts = (“major depressive disorder$”)) and py = (2013–2023)). Fig. 1B displays the list of the top 10 countries in publishing papers on depression. By 2021, the disparity in the total number of publications between China and the USA has been progressively decreasing. After 2022, the number of publications published by researchers in China has surpassed that of the USA (Fig. 1C). Keywords co-occurrence analysis was conducted based on literature sourced from the Web of Science database. This analysis was performed to reflect the distribution of hot topics within the field. As shown in Fig. 1D, the hot research topics in depression are as follows: general symptoms of depression, the risks of depression, the effects of depression, animal models of depression, associated brain regions and functional connections of depression, antidepressants, and depression therapy, comorbidity of depression and other diseases, epidemiology of depression, depression management in primary care, and categories of depression. Establishing appropriate animal models is a fundamental basis for deeply studying the mechanisms and other aspects of depression.

## Diverse Animal Models of Depressive Disorder

**Fig. 2:**
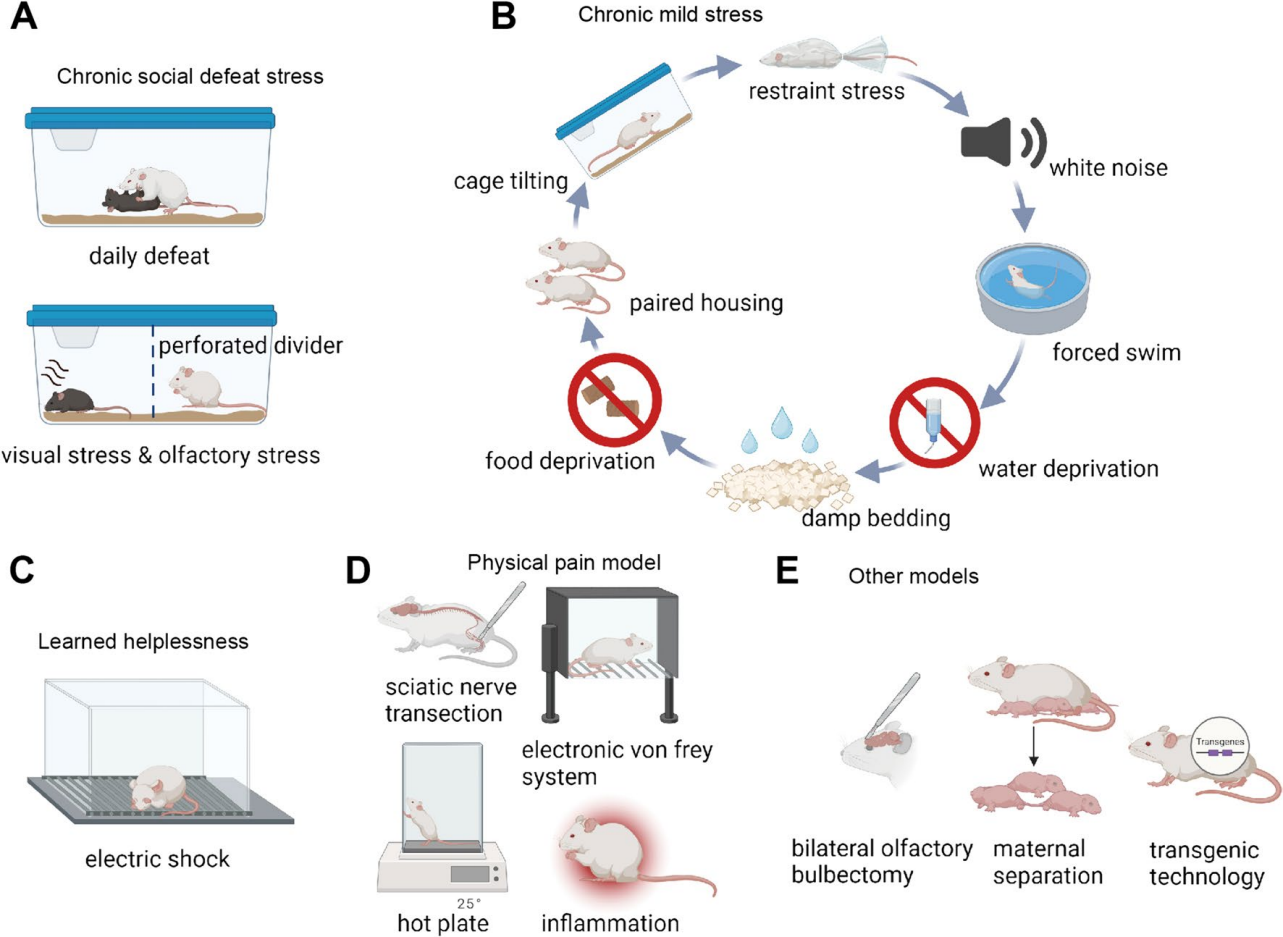
Animal models for the study of depressive disorder. **A** Chronic social defeat stress (CSDS). In this model, depression is induced over 10 days by directly exposing C57BL/6J mice to a larger and more aggressive CD-1 mouse for 5 min per day. **B** Chronic mild stress (CMS). In this model, the mice are exposed to a series of low-intensity stressors at unpredictable times for several weeks. **C** Learned helplessness (LH). The mouse is exposed to unpredictable and inescapable electric foot-shock resulting in a defect in its escape behavior and the manifestation of depressive symptoms. **D** Physical pain model. Transection of the sciatic nerve can result in persistent neuropathic pain, while exposure to stimuli such as a hot plate, electronic von Frey, and inflammatory agents can induce chronic nociceptive pain. These chronic pain conditions are associated with the development of depressive behaviors. **E** Other models. Surgical models, early-life stress, and transgenic techniques are utilized to study depression, such as 5-HTT-/- mice. Schematic figures are created with Biorender.com.

Stress is a well-known precursor to depression in individuals with genetic vulnerability [6, 7]. This established connection between stress and depression has prompted investigators to develop animal models of stress such as social defeat, unpredictable chronic stress, learned helplessness, maternal deprivation, immobilization, isolated stress, sleep deprivation, and forced swim [3, 8, 9]. Fig. 2 shows the current widely used animal model of depressive disorder. We have comprehensively compared the advantages and disadvantages of these depression models to assist researchers in selecting an appropriate animal model for depression studies (Table 1).

**Table 1:** Advantages and disadvantages of depression models.

Animal models	Advantages	Disadvantages
Chronic social defeat stress	Excellent face and construct validity; model social stress	Male and female social defeat models are different; wounds are usually inevitable
Chronic mild stress	Face validity and construct validity	Time-consuming; mild and variable phenotypes; multiple equipment and stimuli;
Learned helplessness	Face, construct, and predictive validity	A short depression duration; species diversity
Physical pain model	Model MDD induced by physical pain; study the perception of pain	Large surgical wounds; cancer pain is very painful

### Chronic Social Defeat Stress (CSDS)

Given that social factors predominantly trigger stress-related neuropsychiatric disorders in humans [10], it's essential to study the effects of social stress using animal models. The CSDS paradigm has been widely used in rodent depression studies, as these animals exhibit some of the cardinal features of human depression like anhedonia, reduced social interaction, attenuated weight gain, increased submissive behavior, and anxiety [8, 11–14]. This paradigm repeatedly exposes animals to a stronger and more aggressive strain, such as CD-1, subjecting them to social defeat. Specifically, the defeated mice after CSDS are divided into susceptible and resilient subtypes, which model the varied human responses to stress [11]. Behavioral changes in defeated mice, such as depression, anxiety, and cognitive impairment, are observed [15].

Historically, Kudryavtseva first reported that subordinate C57BL/6J strain mice exhibited depression-like behavior after defeat in the sensory contact model [16]. Subsequently, researchers continuously standardized the CSDS model. Nestler and colleagues in 2006 reported a standard process for establishing the CSDS mouse model: male C57BL/6J mice are exposed to different CD1 aggressor mice for 10 min daily for 10 consecutive days. After defeat, the resident CD1 mice and defeated C57BL/6J mice are housed in one half of a cage separated by a perforated Plexiglas divider with holes to allow sensory contact for the remaining 24 h after each session. After the final defeat, mice are individually housed with unrestricted access to food and water [14]. In 2011, a standardized protocol was developed, considering various influential factors in the CSDS model, such as the validity of the model, experimenter experience, and differences in individual responses to stress [17].

The advantage of the CSDS model is that it simulates the mechanism of depression-like behavior at the social level and achieves higher structural validity. Unlike other stress models, social defeat represents a unique stressor in terms of the magnitude and quality of the stress response [18]. It more closely resembles human conditions compared to nonsocial stress models like repeated restraint stress [19]. Therefore, the CSDS paradigm is the most commonly used model for studying depression in rodents [20]. However, this model has two main limitations: its symptoms may overlap with those of anxiety, potentially misleading investigators; and it's applicable mostly to male rodents due to the absence of consistent and reliable aggression in female resident-intruder interactions [21].

### Chronic Mild Stress (CMS) and Chronic Unpredictable Mild Stress (CUMS)

As is known, repeated presentation of the same stressor often results in adaptation and may decrease depressive and anxiety-like behaviors [22, 23], a process that can be circumvented by applying a range of stressors in an unpredictable order [1]. Besides, previous research has demonstrated that chronic, uncontrollable stress can impair the brain’s reward system [24]. Thus, the chronic stress animal model was developed to investigate neuropathology [25] and potential therapeutic targets [26, 27] of depressive disorder. The CMS paradigm uses long-term chronic low-level stimulation to continuously expose animals to a series of unpredictable mild stimuli (e.g., small temperature reductions, disruption in the dark–light cycle, changes of cage mates, or dampened bedding), as well as daily random deprivation of water or food for a period of 3 weeks to 3 months [28]. It aims to model the gradual development of a chronic depressive-like state in response to stress, as much as possible similar to the real circumstances of a patient's illness.

Historically, Katz and colleagues developed the first CMS paradigm [29], involving harsh stressors like electric shock, cold swimming, and heat stress [30, 31]. These stressors can cause an increase in plasma corticosterone levels and a reduction in sucrose preference [31, 32], indicating that chronic stress may induce anhedonia. However, this protocol has rarely been used due to serious ethical concerns and unrealistic induction conditions [30–34]. Then, Willner further developed the CMS paradigm [25, 35], focusing on simulating anhedonia by exposing animals to varied mild stressors, such as periods of food and water deprivation, temperature changes, and altering cage mates in an unpredictable manner [36, 37]. This approach more effectively mirrored human life stressors, with sucrose preference reduction reversible by various antidepressants (ADs), including tricyclic antidepressants, selective serotonin reuptake inhibitors, serotonin and norepinephrine reuptake inhibitors, monoamine oxidase inhibitors, and atypical ADs [38]. This protocol of CMS has since been extensively described and used [39].

The advantages of the CMS model are face validity and construct validity. This model results in enduring changes across behavioral, neurochemical, neuroimmune, and neuroendocrinological parameters, mirroring dysfunctions seen in depressed patients [25, 39, 40]. Therefore, it is considered one of the most extensively validated and realistic models of depression. However, establishing this paradigm in a new lab is challenging, and replicating data across labs is sometimes difficult, perhaps due to its time-consuming protocol, as well as mild and variable phenotypes [40]. Numerous high-frequency protocols also require extensive experimental space, which may also result in low efficiency.

### Learned Helplessness (LH)

Helplessness, a core symptom of MDD, is extensively studied in both clinical and preclinical depression research. The LH paradigm, one of the earliest animal depression models [41], induces a depressive-like state through uncontrollable and unpredictable electrical foot-shock stress [42–44]. The classic experimental procedure is a triadic design with two control groups: the first control group experiences controllable shocks (like escaping, lever pressing, or wheel turning) [45, 46]. The second group, yoked to the first, receives the same shocks but as unpredictable and uncontrollable. The third group is an unstressed control group. This procedure allows comparison across groups, highlighting that the key factor in stress-induced deficits is the uncontrollability, not the stress itself [47].

Historically, LH was first reported in the 1960s by Richard L. Solomon, who was investigating the separability of classical Pavlovian conditioning and instrumental learning. He discovered that prolonged exposure to uncontrollable traumatic events led to unexpected behavioral changes. Overmier and Seligman later found that short, distributed exposure to uncontrollable trauma over several hours caused significant deficits in behavioral coping, associative learning, and emotional expression, a phenomenon they termed “learned helplessness” [48]. Helpless animals showed sustained changes such as weight loss, sleep pattern disruptions, altered hypothalamus-pituitary-adrenal (HPA) axis activity, and hippocampal spine synapse loss [49, 50]. These behaviors are consistent with those clinically observed in human patients.

The advantage of the LH model is that its symptoms closely resemble those of major depression, most of which are reversible by several acute antidepressant treatments (typically for 3-5 days) [51]. It has a good face, construct, and predictive validity, and the model has been instrumental in confirming various pathophysiological theories of depression [52, 53]. A major limitation, however, is the short-lived nature of depression-like symptoms after exposure to uncontrollable shocks [54]. Besides, susceptibility to LH varies among different strains: the Kyoto and Charles River Holtzmann lines are the most susceptible, and Harlan Sprague–Dawley exhibit moderate susceptibility, while Lewis, Brown Norway, Fischer F-344, and Sasco Holtzman show almost no susceptibility to the effects of inescapable shock [55].

### Physical Pain Model

Physical pain, particularly neuropathic and nociceptive types, is another major contributor to depression [56, 57]. According to the literature, pain resulting from sensory nerve damage can impact depressive moods and lead to neuronal death in brain regions associated with depression, such as the insular lobe, prefrontal cortex, thalamus, hippocampus (HPC), anterior cingulate, and amygdala [58]. Furthermore, chronic pain and depression frequently co-occur [59]. The mechanisms underlying the connection between pain and depression are complex [60, 61].

Physical pain is classified into neuropathic, nociceptive, postoperative, and cancer pain, among others, organized by type of pain. Neuropathic pain specifically arises from a lesion or disease within the somatosensory system itself [62] and occurs due to injuries or chemical exposure. Neuropathic pain models involve surgical transection of specific nerves, like the sciatic nerve [63–65] or spinal nerves [66, 67], while preserving surrounding tissue for histologic examination. In addition, inflammation secondary to a crushing injury can also cause neuropathic pain, as reported in models like spinal nerve ligation at L5-L6 [66, 68], partial saphenous nerve ligation [69], and full or partial sciatic nerve ligation [70–73]. Nerve ligation models effectively replicate aspects of human neuropathic conditions, such as sciatica and other constrictive nerve injuries.

Nociceptive pain activates pain receptors that detect stimuli and transmit signals to the central nervous system for recognizing and reacting to injury or potential harm [74]. Nociceptive pain models are based on spinal reflexes with assessments including tail flick, paw withdrawal, paw lifting, flinching, guarding, and licking [75]. The types of nociceptive stimuli can be categorized into thermal (hot plate or radiant heat for paw/tail stimuli), mechanical (von Frey filaments and Randall-Selitto paw pressure tests for pressure application), and chemical stimulation (injecting irritants like formalin, capsaicin, or acetic acid). Inflammatory models [75, 76], such as carrageenan-induced edema in rat hind paws [77] or Complete Freund’s adjuvant for inflammation [78], are also used. These models help study the perception and sensitivity of the organism to pain stimuli, evaluate the efficacy of analgesics in nociceptive pain, and explore potential therapeutic targets for pain relief.

Postoperative nociceptive pain can lead to persistent nociplastic pain states after surgery, causing depression and increased suffering in patients. To model this, animals undergo similar surgical procedures, such as the plantar incision model [79–81], which involves a hind paw skin incision and can include deeper tissue manipulation by retracting, stretching, or incising the plantaris muscle in the arch of the paw. For cancer pain, the degree of pain is tied to the tumor microenvironment. Bone and pancreatic cancers are very painful, while others like lipomas and melanomas may not cause pain. In rodents, osteolytic bone cancer pain can be modeled by injecting osteolytic fibrosarcoma cells into the femur [82, 83], humerus, calcaneus [84], or tibia [85]. In addition, a transgenic mouse spontaneously expressing exocrine pancreatic tumors is used to model pancreatic cancer [86].

### Other Models

In addition to the above models, in the recent decade, several modeling methods like surgical models, early-life stress models, and mutant approaches have been proposed. Bilateral olfactory bulbectomy (OBX), has been employed as a surgical model of depression, affecting endocrine, immune, and neurotransmitter systems [87]. In rodents, the olfactory system, a part of the limbic region involving the amygdala and HPC, influences behavior and emotions. Post-OBX, rodents exhibit hyperactivity, social behavior changes, increased nocturnal activity, learning and memory deficits, and altered taste-aversion [88]. These changes are thought to stem from dysfunctions or compensatory actions in the cortical → hippocampal → amygdala circuits, similar to areas affected in major depression [89]. Chronic antidepressant treatment has been shown to address many of the changes induced by OBX [88]. While OBX has a face and predictive validity, it lacks etiological and construct validity.

Numerous experimental methods have been established to induce early-life stress in rodents during crucial developmental stages [90, 91]. Maternal separation serves as a significant model for studying the pathophysiology and treatment of major depression. For instance, the administration of ADs in adult male rats undergoing maternal separation can normalize their anxiety-like behavior, endocrine stress response, and ethanol preference [92]. A recent development is the chronic early-life stress model, which has both immediate and lasting impacts on the HPA system and cognitive functions. This model involves disrupting normal interactions between a mother and her offspring, leading to impaired hippocampal learning and memory functions and decreased survival of adult neurons [93, 94].

The advancement of genetic techniques has rendered mutant models highly effective for identifying potential targets of depression. In the last few years, various mouse lines have been created to investigate depression-related genes, implicated in depression, aligned with theories on monoamines, neurotrophins, and the HPA axis [95–97]. For instance, mice lacking the 5-HT transporter (5-HTT^-/-^) display anxiolytic and antidepressant-like responses in behavioral tests like the elevated plus maze, tail suspension test, and forced swim test [98–100].

Although numerous animal models of depression, each with its own predictive, face, and construct validity, have significantly enhanced our understanding of the neurobiology of depression, they come with several limitations. Importantly, the exploration of specific neural circuits involved in depression represents a new frontier in advancing the treatment of this complex disorder.

## Neural Circuits of Depressive Disorder

**Fig. 3:**
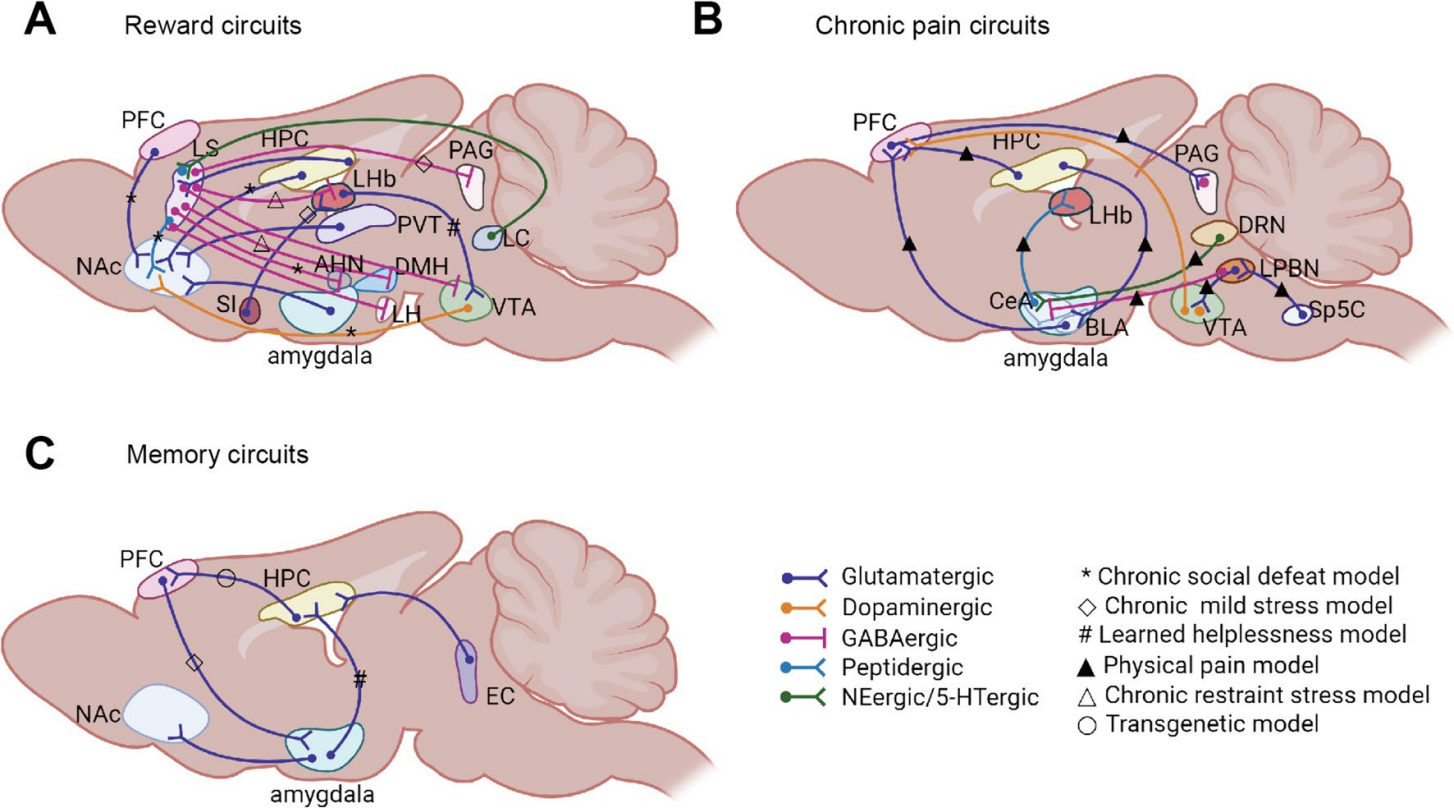
Schematic of the major neural circuit connections involved in regulating depression-related behaviors. The figure shows only a subset of the many known interconnections among various brain regions. The symbols on the connecting lines signify the categories of animal models used for exploring this pathway. **A** Reward circuits. **B** Chronic pain circuits. **C** Memory circuits. PFC, prefrontal cortex; NAc, nucleus accumbens; LS, lateral septum; HPC, hippocampus; LHb, lateral habenula; VTA, ventral tegmental area; PVT, paraventricular thalamus; PAG, periaqueductal gray; SI, substantia innominata; AHN, anterior hypothalamic nucleus; LH, lateral hypothalamus; DRN, dorsal raphe nucleus; LPBN, lateral parabrachial nucleus; BLA, basolateral amygdala; CeA, central amygdala; Sp5C, spinal trigeminal subnucleus caudalis. Schematic figures are created with Biorender.com.

Several brain regions and circuits, notably within the interconnected limbic system, play key roles in regulating emotion, reward, and executive function. Dysfunctions in these regions are linked to depression and anti-depressant action. Classically, the ventral tegmental area (VTA) is central to reward processing, the nucleus accumbens (NAc) to hedonic and motivational deficits associated with depression, the amygdala to fear, and the HPC to cognitive impairments. The lateral habenula (LHb) is thought to influence anhedonia [101]. Here we primarily review a brain network (Fig. 3) to elucidate how specific circuits regulate depressive behavior.

### Depression and Reward

The complexity of the reward function is due to the involvement of a common circuit linking the dopaminergic midbrain, basal ganglia, and frontal cortex in many reward processes [102]. There is growing evidence of the brain’s reward circuitry playing a crucial role in the development and symptoms of depression [103]. The concept that midbrain dopamine (DA) systems influence depression-like behaviors emerged from studies using DA receptor antagonists [104]. Stress, particularly in animal models of depression, activates VTA DA neurons, enhancing dopaminergic transmission to limbic targets like the NAc. Antidepressants alter this dopaminergic activity, and experimental manipulation in the VTA → NAc dopaminergic pathway affects depression-like behaviors in rodents under acute stress. Specifically, optogenetic activation of VTA DA neurons during CSDS exacerbates depressive phenotypes [105], while optogenetic inhibition of VTA-NAc DA neurons alleviates the anhedonia induced by CSDS [106].

The ventral striatum (vSTR) is crucial in initiating reward-related behaviors and managing social stress [107, 108]. Previous research has shown that intralaminar thalamus (ILT) and PFC projections to the vSTR regulate reward behaviors [109, 110], but the specific inputs responsible for mediating chronic stress effects remain uncertain. Christoffel *et al.* [111] explicitly illuminated the vSTR's unique role in stress-related synaptic remodeling and behavior. This underscores how synaptic alterations in the vSTR, particularly at ILT glutamatergic inputs to vSTR medium spiny neurons, are key in stress susceptibility following CSDS. The NAc, as a region of the vSTR, is pivotal in processing rewarding stimuli. Lind *et al.* [112] investigated NAc shell input-specific reward behaviors, using spatially dependent optogenetic self-stimulation tasks to assess mouse responses. They confirmed distinct roles for various pathways to the NAc shell: the medial prefrontal cortex (mPFC) → NAc shell pathway in facilitating place preference, the vHPC → NAc shell pathway in consistently promoting place preference, the basolateral amygdala (BLA) → NAc shell pathway in generating modest place preference linked to time sensitivity, and the paraventricular thalamus (PVT) → NAc shell pathway in reducing but not fully negating, place preference. Besides, Vollmer *et al.* [113] explored the role of PVT → NAc in reward seeking. Their work revealed this pathway is crucial in suppressing sucrose self-administration through NAc parvalbumin (PV) interneurons and Ca^2+^-permeable AMPA receptor-rich synapses, with opioid intervention quickly changing this behavior.

The ventral HPC (vHPC) is recognized for encoding reward-predictive stimuli and influencing reward-oriented behaviors [114–118]. Hippocampal synapses on the NAc exhibit high plasticity, with brief, high-frequency activity inducing long-term potentiation (LTP) and sustained reward responses. Chronic stress has been shown to cause anhedonia, weaken these synaptic connections, and impair LTP, effects that can be reversed by antidepressants [115]. Chronic stress also leads to dendritic spine atrophy in hippocampal CA1 and CA3 pyramidal cells, as well as a reduction in neurogenesis [119]. Conversely, enhanced neurogenesis in the hippocampus of a transgenic mouse can reduce anxiety and depression-like behaviors [120]. In addition, cocaine exposure has been linked to strengthened HPC–NAc connections [121]. Bagot *et al.* [122] utilized optogenetic techniques to manipulate specific synaptic functions and discovered that glutamatergic vHPC projections to the NAc modulate the behavioral effects of CSDS.

The LHb, a brain region involved in depression, plays a key role in processing both reward and punishment [123–126]. Activation of LHb neurons has been recorded in response to nonrewarding or unpleasant events. Notably, acute stress alters LHb's response, turning reward signals into ones resembling punishment, which is linked to depression [127]. Research has highlighted that attenuating LHb hyperactivity can effectively lessen depressive symptoms. Inputs from the basal forebrain (BF), particularly the substantia innominata (SI), a BF subregion, are instrumental in relaying punishment and reward signals to the LHb [128, 129]. Previous research has shown that selective activation of glutamatergic SI neurons results in real-time conditioned place aversion, while activation of its GABAergic neurons leads to conditioned place preference (CPP) [130]. Cui *et al.* [131] demonstrated that chronic activation of the SI → LHb circuit induces depressive-like behaviors, whereas its inhibition alleviates stress-induced depressive-like behaviors. In addition, reward consumption can buffer depressive-like behaviors. A study on depression models discovered that glutamatergic LHb neurons that project to the VTA receive increased dopaminergic inputs, potentially causing aversion and affecting cognitive functions in major depression [124]. Clinical evidence has also shown that deep brain stimulation inactivates the habenula, leading to complete remission of treatment-resistant major depression in patients [132].

The lateral septum (LS), a forebrain structure, participates in various behavioral responses to stress and also plays a crucial role in reward processes by eliciting intrinsic rewarding properties [133, 134]. This insight originates from pioneering self-stimulation studies [135, 136]. A recent study has revealed that the CA3 → LS → VTA pathway mediates cocaine-seeking behaviors [137]. Chemogenetic inhibition of the dorsal CA3 → LS pathway has been found to reduce cocaine-seeking behavior [138]. Further research indicates that the LS, particularly its rostral part, activates lateral hypothalamic orexin neurons during cocaine CPP. This activation is essential for the expression of cocaine preference [139]. In addition, LS neurons expressing neurotensin (NT^LS^) and somatostatin (Sst^LS^) are hyperactivated under stress in mice. Our group investigated NT^LS^ neurons, uncovering that social reward functions are impaired in the CSDS depression model [140]. Our work employed a range of methods, including *in vivo* imaging and chemogenetics manipulations, demonstrating the necessity and sufficiency of activating the NT^LS^ → anterior hypothalamic nucleus and NT^LS^ → NAc circuits in altering social investigation and preference behaviors post-social trauma. An *et al.* [134] focused on Sst^dorsal LS^ neurons, finding that they are responsive to various stressors, and their activity is influenced by norepinephrine of the locus coeruleus (LC). The LS consists primarily of GABAergic neurons. The GABAergic LS neurons’ projections to the dorsal periaqueductal gray (dPAG) are associated with depression-related behaviors in the chronic unpredictable stress model. Activation of the LS → dPAG circuit reduces sucrose preference in mice, whereas its suppression induces an antidepressant effect [141]. The latest research on LS has pinpointed a subset of GABAergic adenosine A_2A_ receptor-positive LS neurons that contribute to depressive symptoms. These neurons exert their influence through direct connections to both the LHb and dorsomedial hypothalamus [142].

### Depression and Chronic Pain

Chronic pain and depressive symptoms, often encountered clinically [143, 144], have been found to mutually exacerbate each other [145, 146]. Shared brain regions in these conditions include the amygdala and the dorsal raphe nucleus (DRN). Zhou *et al*. [147] elucidated a neural circuit for comorbid depressive symptoms in chronic pain. The 5-HT-expressing neurons in the DRN (5-HT^DRN^) project to somatostatin (SOM) expressing neurons in the central amygdala (CeA, referred to as the ‘nociceptive amygdala’), and the LHb is the output of the 5-HT^DRN^ → SOM^CeA^ circuit. As for this circuit function, they demonstrated that inhibiting the 5-HT^DRN^ → SOM^CeA^ → LHb pathway produces depression-like behavior, whereas its activation alleviates pain and depression-like symptoms. In addition, Zhang *et al.* [148] reported a neural network linking the spinal trigeminal subnucleus caudalis (Sp5C) to the lateral parabrachial nucleus (LPBN) and the VTA DA neurons, linked to depression in chronic neuropathic pain. They discovered that glutamatergic projections from the Sp5C innervate the glutamatergic neurons in the LPBN, which project to the VTA and modulate the activity of the VTA DA neurons. Upon activating the Sp5C → LPBN → VTA circuit, there is an increase in the firing activity of the VTA DA neurons. Conversely, inhibiting this elevated activity in the VTA DA neurons effectively reverses depressive behaviors, thus “uncoupling” chronic pain from comorbid depression.

The mPFC, critical in the affective processing of pain, undergoes plasticity during chronic pain progression. Several studies have demonstrated decreased activity in layer V pyramidal neurons (PNs) of the prelimbic (PL) mPFC in rodent models of chronic pain. Optogenetic activation of these neurons can alleviate symptoms of sensory and chronic pain [149, 150]. Recently, Huang *et al.* [151] established the significance of the BLA → mPFC → PAG → spinal cord pathway in the development of mechanical and thermal hypersensitivity following peripheral nerve injury. This pathway influences pain behaviors by reducing the noradrenergic and serotoninergic modulation of spinal signals. Subsequently, they further combined optogenetics with behavioral analysis in neuropathic pain models, focusing on the modulation of pain responses by DA projections from the VTA to the mPFC [152]. Moreover, DA was found to augment the activity of neurons projecting from the mPFC layer V PNs to the ventrolateral PAG, resulting in analgesia. At the functional level, fMRI studies in chronic pain patients have identified both hyper- and hypoactivity in the mPFC [153, 154], possibly due to different plasticity across distinct mPFC subregions and cells.

The amygdaloid complex consists of two divisions that are particularly relevant to pain: the BLA and the CeA. The BLA plays a well-established role in integrating polymodal sensory information, and by transmitting this information to the CeA, facilitates injury or pain avoidance, which are subsequently executed by brainstem centers. Apart from the CeA, the mPFC serves as another important output target of the BLA for regulating pain perception [155]. Previous seminal work from Ji *et al.* [156] has shown that chronic pain states result in BLA neuronal hyperactivity, causing mPFC deactivation and associated cognitive deficits. This occurs through feed-forward inhibition facilitated by PV-type GABAergic neurons in the PL cortex, activated by BLA inputs [150]. Ji and colleagues also found that metabotropic glutamate receptors 1 (mGluR1) and 5 (mGluR5) in the PFC play a significant role in regulating the inhibitory effect of BLA input. Specifically, mGluR1 receptors inhibit feedforward inhibition by dampening the excitability of PNs in the PFC, while the mGluR5 receptors increase PFC activity by enhancing endogenous cannabinoid release [157, 158]. A recent study has suggested that the CeA can bidirectionally modulate nociception [159], with neurons expressing protein kinase C-delta enhancing pain-related responses, and neurons expressing somatostatin (CeA-SOM) can drive antinociception. This dual and opposing function of the CeA arises from changes in GABAergic neuron excitability, driven by PBN excitatory inputs. The CeA also influences the interplay of chronic pain and anxiety. Zussy *et al.* [160] demonstrated that mGlu4 activation in the CeA not only abolishes inflammatory mechanical allodynia but also reduces anxiety and depression-like symptoms, reflecting the multifaceted role of the CeA in pain and emotion regulation.

Amygdala activity has been suggested to contribute to inflammatory processes [161]. Zheng *et al.* [162] reported in an LPS-induced neuroinflammation mouse model that microglial activation and pro-inflammatory cytokine production in the lateral amygdala, along with an increase in presynaptic glutamate release, leads to an excitatory/inhibitory imbalance. Mice exhibit anxiety and depression-like behavior. Interestingly, the anti-inflammatory factor IL-10 can mitigate these effects by normalizing gamma-aminobutyric acid (GABA) transmission in the amygdala, reducing anxiety-like behaviors and substance dependence [163]. Similarly, in adult male Sprague-Dawley rats, social defeat experiences enhance microglial activation and neuronal firing in the BLA, contributing to anxiety-like behaviors. Blocking microglial activation in these instances prevents such behaviors [164], suggesting a reciprocal relationship between amygdala activity and inflammation, and their joint contribution to anxiety.

The HPC is one of the brain regions involved in the regulation of pain signals, being active during the processing of pain and the modification of nociceptive stimuli. Numerous animal studies have linked elevated pro-inflammatory factors in the HPC to stress-induced anxiety and depressive-like behaviors [165]. For instance, acute stress significantly increases TNF-α production by the hippocampal microglia in mice [166]. Cytokines like IL-1β and TNF-α can suppress neurogenesis in the dentate gyrus (DG) [167], leading to neuronal apoptosis [168] and heightened anxiety-related behavior [169]. Oral administration of minocycline, a microglia inhibitor, alleviates hyperanxiety in mice [170]. In terms of neural circuits, recent research has revealed that the vCA1 → BLA and vCA1 → infralimbic cortex (IL) pathways play distinct roles in chronic inflammatory pain at different stages [171]. The vCA1 → BLA pathway appears to be associated with early-stage pain perception, whereas the vCA1 → IL pathway may be involved in pain recovery during the more complex stages.

### Depression and Memory Deficit

Memory, a fundamental aspect of human and social behavior, encompasses the processes of encoding, storing, and retrieving social cues [172]. This complex function allows individuals to accurately recall past social interactions, facilitating appropriate social responses, and continuous memory updating with each new encounter. However, impairments in cognitive and memory functions can disrupt a person’s ability to regulate emotions [173, 174]. In particular, brain regions like the left dorsolateral PFC, the dorsomedial PFC, and the anterior cingulate cortex, critical for cognitive control, are also implicated in the abnormal utilization of emotion regulation strategies [175]. This includes a tendency towards the prolonged use of maladaptive strategies and challenges in the effective implementation of adaptive strategies [176]. Such memory deficits are common symptoms in patients with depression, including verbal delayed memory, visuospatial memory, verbal working and long-term memory, and working memory [177–180]. They can lead to increased suicidal tendencies, presenting serious clinical and social impacts [181, 182]. The awareness level of memory deficits is an important criterion for measuring the severity of depression [183].

A prevailing theory suggests that dopaminergic signaling plays a role in the development of memory deficits in depression. In animal models, depressive-like states are associated with alterations in dopaminergic neurotransmission, including changes in dopamine levels, tyrosine hydroxylase activity, and dopamine receptor expression [184]. Rats subjected to chronic restraint stress [185] or unpredictable CMS [186, 187] exhibit reduced dopamine levels in the PFC and HPC, which are accompanied by memory impairments. These changes can be reversed by Shen Yuan Gan and taurine [186, 187]. Temporal object memory deficits, found in the maternal separation model, can be restored by administering a D1 agonist [188]. Furthermore, patients with depression also exhibit impairments in positive memory. The release of dopamine in the HPC is essential for memory consolidation [189, 190]. Taken together, memory deficits in depressed patients may occur through the impact on HPC and dopaminergic midbrain regulation of memory consolidation [191].

The HPC is involved in managing various cognitive and mnemonic impairments induced by stress [192, 193]. It has been demonstrated that stress can inhibit hippocampal neurogenesis [194, 195]. In patients with depression, both a reduction in hippocampal neurogenesis and a decrease in hippocampal volume have been reported [196]. Various antidepressant treatments have been shown to stimulate neurogenesis in adult HPC [197]. Adult rats exposed to prenatal restraint stress exhibit a reduction in glutamate release in the vHPC, resulting in impaired social memory and depressive-like behaviors. These effects can be reversed with treatments such as agomelatine or fluoxetine [198]. In addition, dCA2 silencing impairs social memory [199]. Methods used to inactivate the dCA2 region, such as excitotoxic lesions and tetanus neurotoxin expression, result in its permanent silencing [200, 201]. This irreversible action might lead to lasting reactive changes in brain areas beyond dCA2, potentially contributing to socio-cognitive deficits. Phillips *et al.* [202] have confirmed that prolonged activation of mPFC-projecting vHPC neurons impairs social memory. Conversely, their sustained inhibition can rescue social memory deficits in autism spectrum disorder Rett syndrome mice, underscoring the necessity of balanced vHPC → mPFC signaling for social memory recall. Furthermore, the vHPC, receiving inputs from the medial EC, is vital for consolidating memories linked to anxiety and emotional behavior [203, 204]. Zhu *et al.* [204] implicated vHPC glutamatergic neurons in contextual fear memory consolidation.

The BLA is widely recognized for its critical function in handling emotions and motivation, significantly contributing to positive and negative emotion-related events [205, 206]. Importantly, the BLA is intricately connected with the HPC [207]. Emotional arousal activating the BLA impacts memory-related functions in the HPC. Studied have demonstrated that the BLA regulates LTP in the HPC DG [208], whereas BLA lesions inhibit the effect of glucocorticoid on modulating spatial memory in the dHPC [209]. Yang *et al.* [210] highlighted the importance of the BLA → HPC circuit in linking emotional states to spatial memory. Their finding indicated that LH attenuates the activity of the posterior BLA (pBLA) → vCA1 circuit, leading to memory deficits. In contrast, learned hopefulness strengthens this connection, thereby improving spatial memory. Stimulating this circuit effectively reverses the memory deficits induced by LH, enhancing synaptic transmission and dendritic plasticity in the CA1 region. This enhancement is facilitated by increased cAMP-response-element-binding protein (CREB) expression and a rise in intrasynaptic AMPA receptors. Pi *et al.* [211] employed the Barnes maze (BM) to demonstrate that anxiety status is associated with spatial memory deficits in mice with neuropsychiatric or neurodegenerative disorders, such as Alzheimer’s disease. Stimulating the glutamatergic inputs from pBLA to vCA1 reduces anxiety behaviors and spatial memory impairments, dependent on calbindin1 (Calb1) in the vCA1. Recently, another study has shown that optogenetically reactivating DG engram cells formed after a natural rewarding experience can acutely reduce depression-related behaviors. This effect is mediated by glutamatergic transmission from the amygdala’s axonal terminals to the NAc shell, essential for the real-time antidepressant effects of these reactivated DG engram cells [193].

The role of the mPFC in emotional behavior regulation is well-characterized, both negative and positive [212–214]. The IL PFC is essential for the extinction of memory. Chemogenetic inhibition of the IL →BLA pathway impairs the formation of extinction memory [215]. Yu *et al.* [216] developed a new go/go task to test the memory responses to ambiguous cues in mice. This work revealed that activation of pBLA *Calb1*–positive neurons is involved in the generalization of reward memory. Functionally, stimulating the IL to the pBLA projection significantly enhances the reward memory generalization and inhibits anxiety and depressive behaviors in mice subjected to unpredictable CMS stress. Studies have shown that silencing the mPFC neurons prevents the antidepressant effects of ketamine, whereas activation of *CaMKII2α*-expressing PNs in the mPFC can mimic these effects [217]. Hare *et al.* [218] demonstrated that the response to ketamine requires *Drd1*-expressing PNs in the mPFC, and stimulating mPFC *Drd1* projections to the BLA can induce antidepressant effects. In preclinical studies of depression, working memory impairment is associated with decreased activity in *Drd1*-expressing PNs in the mPFC [219].

Studies indicate that mice exposed to social stress exhibit impaired memory and alterations in certain biochemical indicators. Specifically, Patki *et al.* used a modified resident-intruder model to induce social defeat stress, finding alterations in BDNF, ERK1/2, IL-6, glyoxalase-1, glutathione reductase-1, Ca^2+^/calmodulin-dependent protein kinase type-IV, and CREB levels in the HPC. These alterations lead to oxidative stress-induced anxiety, depression-like behaviors, and memory deficits [220]. Liu *et al.* [221] demonstrated that unpredictable CMS impairs spatial memory and decreases dendritic spine density, which can be prevented with paeoniflorin—an antidepressant-like herbal medicine. Maternal separation models impair spatial memory and reduce BDNF levels [222–224]. Similar results have been reported in other depression models, including restraint stress [225], chronic immobilization stress [226], LH [227], OBX [228, 229], corticosterone administration [230], LPS injections [231], and a post-stroke depression model [232].

## Conclusion and Future Perspectives

In a retrospective review of previous research, we have identified the advantages and differences among various models commonly used in depression studies. In addition, we have summarized the neural circuits associated with depression. These models provide us with a platform to investigate depressive-like behaviors and physiological changes, and the study of neural circuits helps reveal the neurobiological basis of depression. However, there are still many limitations; for example, current models fail to mimic the relapsing-remitting pattern typical of depression, and there is a notable lack of models focusing on female animals. On the other hand, the development of these animal models for depression is predominantly driven by the needs and objectives of the pharmaceutical industry. Therefore, this expects researchers to undertake more profound and comprehensive research.

In the future, research will need to focus on more refined animal models and the intricacies of the nervous system and more decision-making-based models to identify other features of depression. Gaining a thorough understanding of the behaviors and behavioral characteristics exhibited by mouse depression models, along with advanced neural imaging and modulating techniques like genetically engineered sensor imaging, optogenetics, and deep brain stimulus, will help reveal subtle changes in neural circuits. Research on individual differences will also be a key focus, specifically exploring variations at the neural circuit level among different rodent individuals to better replicate the diversity observed in human depression. Collectively, the development of new strategies and advanced technologies, and the continual demand in neuroscience research for novel tools, will ultimately promote rapid and significant advancements in this field.
